# Data-driven prediction of chemically relevant compositions in multi-component systems using tensor embeddings

**DOI:** 10.1038/s41598-024-85062-z

**Published:** 2025-01-09

**Authors:** Hiroyuki Hayashi, Isao Tanaka

**Affiliations:** 1 Department of Materials Science and Engineering, Kyoto University, Sakyo, Kyoto, 606-8501 Japan; 2 Nanostructures Research Laboratory, Japan Fine Ceramics Center, Nagoya, 456-8587 Japan

**Keywords:** Design, synthesis and processing, Information technology

## Abstract

The discovery of novel materials is crucial for developing new functional materials. This study introduces a predictive model designed to forecast complex multi-component oxide compositions, leveraging data derived from simpler pseudo-binary systems. By applying tensor decomposition and machine learning techniques, we transformed pseudo-binary oxide compositions from the Inorganic Crystal Structure Database (ICSD) into tensor representations, capturing key chemical trends such as oxidation states and periodic positions. Tucker decomposition was utilized to extract tensor embeddings, which were used to train a Random Forest classifier. The model successfully predicted the existence probabilities of pseudo-ternary and quaternary oxides, with 84% and 52% of ICSD-registered compositions, respectively, achieving high scores. Our approach highlights the potential of leveraging simpler oxide data to predict more complex compositions, suggesting broader applicability to other material systems such as sulfides and nitrides.

## Introduction

The discovery of novel materials not only enhances our understanding of fundamental physical mechanisms but also accelerates the development of new functional materials. Although newly discovered materials do not always exhibit superior properties, materials with similar compositions or crystal structures may lead to the discovery of new materials with excellent properties^1–8^. For example, in perovskite-type oxides, structural similarity has contributed to the discovery of new materials with high electrical and catalytic properties^9,10^.

Multi-component materials attract significant interest in various application fields, particularly in energy materials, catalysts, and electronic materials^11–16^. Therefore, efficient exploration of these materials is one of the key challenges. In recent years, advances in computational materials science have enabled the generation of virtual chemical compositions by substituting constituent elements in newly discovered materials based on their crystal structures, allowing for high-precision calculations of various physical properties^17–19^. However, multi-component materials remain underexplored, and the number of known crystal structures is limited. As a result, the discovery of new materials is also crucial for computational materials science.

A challenge in exploring multi-component materials is the vast number of possible combinations of elements and composition ratios, leading to a broad search space. While combinatorial experiments and automated robotic experiments have been researched for more efficient synthesis^20–24^, the current level of efficiency is insufficient given the expanding search space. Conducting exhaustive experiments across such a wide space can lead to wasted effort and resources, making it necessary to develop a system that can predict compositions with high synthesizability. In previous research by the authors^25,26^, a method was developed to predict Chemically Relevant Compositions (CRC) using tensor decomposition. However, because the dimensionality of the tensor changes depending on the number of constituent elements, separate prediction models were needed for simple and multi-component compositions. This resulted in lower predictive performance for multi-component compositions, especially when the number of known data points was limited relative to the search space. Additionally, while methods exist to vectorize chemical compositions using features like atomic numbers and electronegativities of the constituent elements^27–29^, using tensor embedding vectors obtained through tensor decomposition is expected to provide representations that more directly correlate with the presence or absence of chemical compositions.

**Fig. 1:**
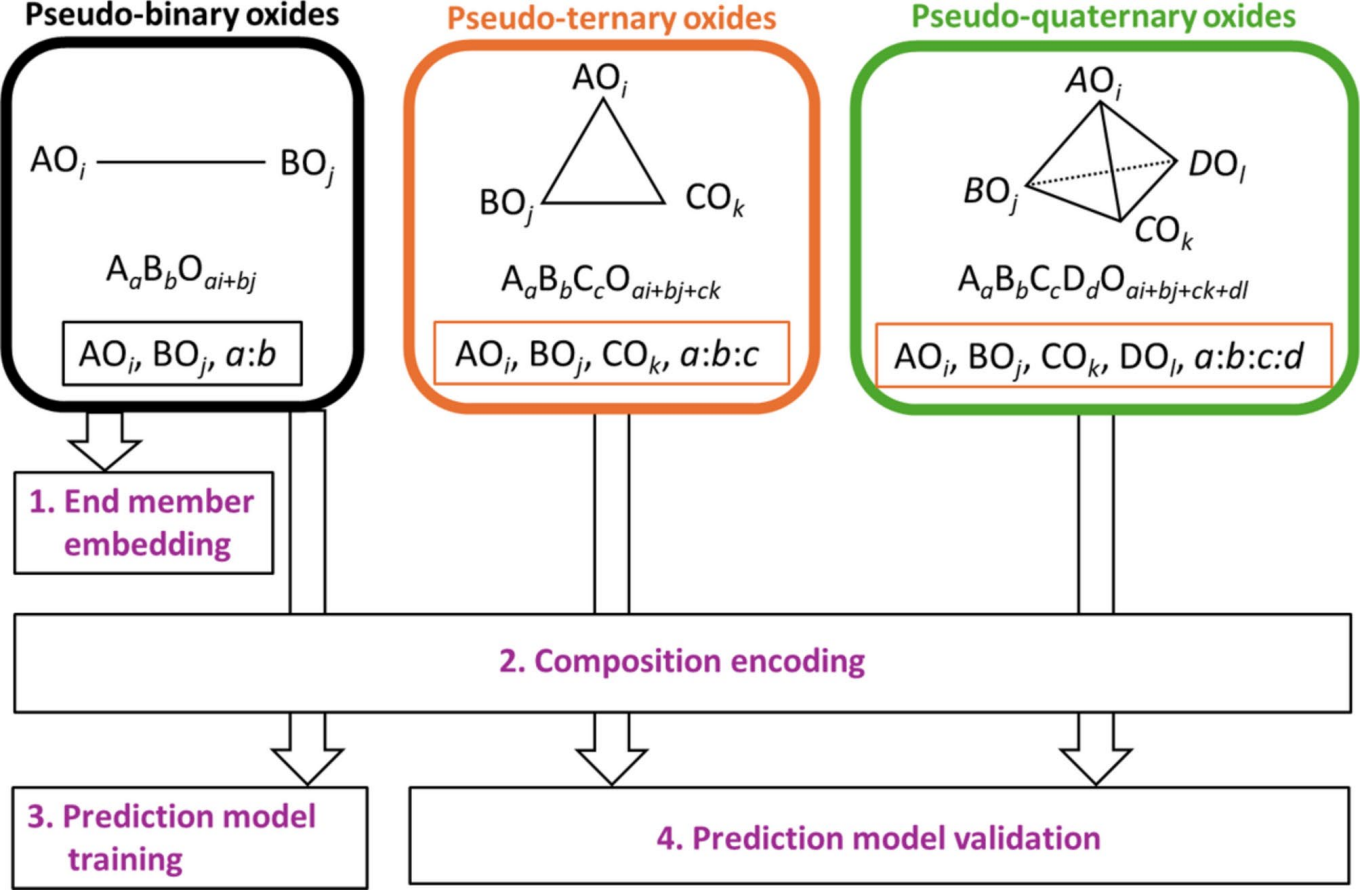
Overview of the proposed method. The target data consists of pseudo-binary, pseudo-ternary, and pseudo-quaternary oxide compositions extracted from the ICSD. These compositions are transformed into data representing the end members and their compositional ratios. First, Tucker decomposition is applied to the pseudo-binary oxide data to obtain tensor embeddings of the end members. Next, the obtained tensor embeddings are used to encode each composition into a compositional descriptor. A classification model is then trained using the encoded pseudo-binary oxide data, based on whether the compositions are registered in the ICSD. Finally, the trained classification model is used to predict the registration of encoded pseudo-ternary and pseudo-quaternary oxide compositions in the ICSD, and the predictions are evaluated against actual ICSD registrations.

**Fig. 2:**
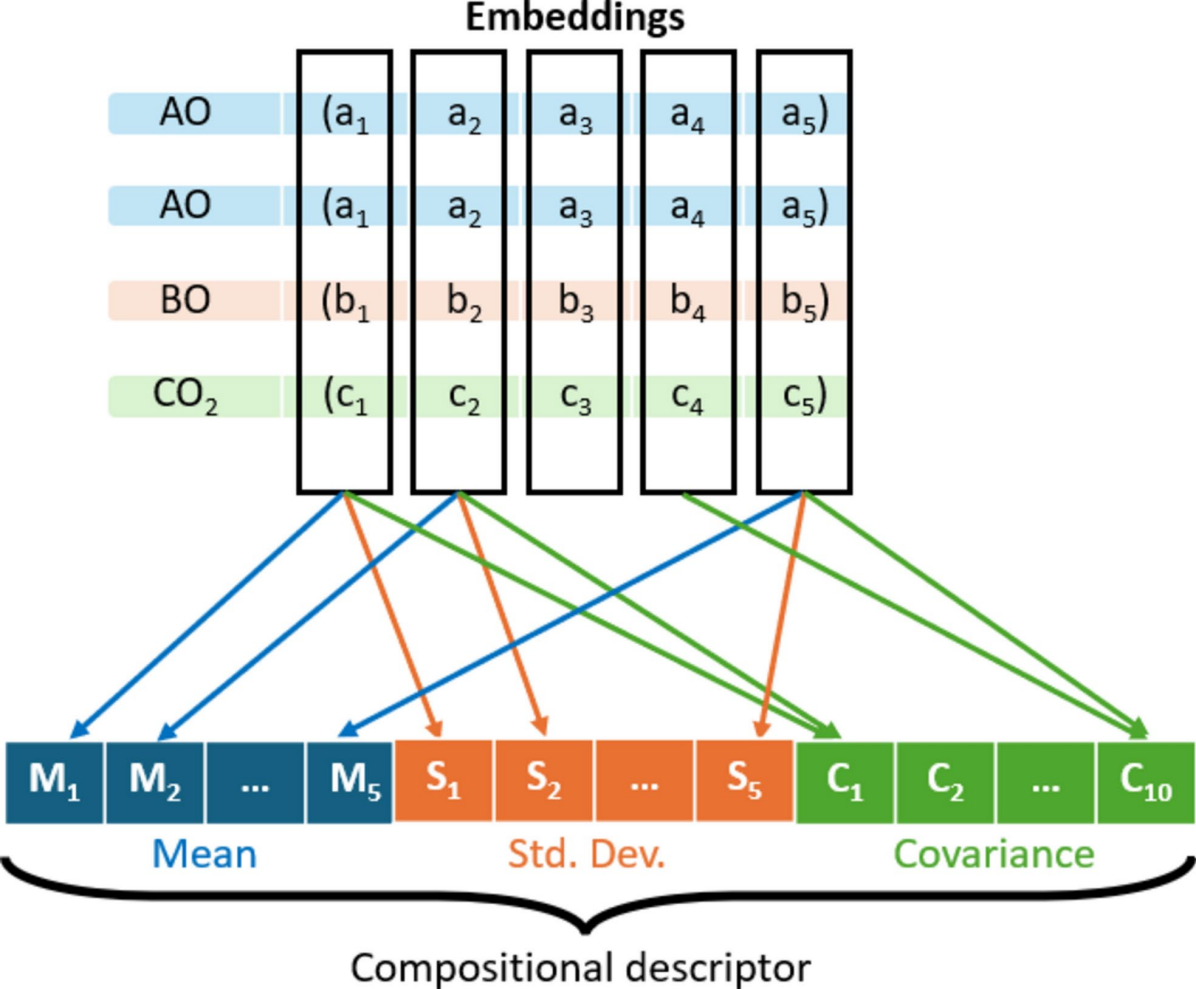
This figure illustrates the method for encoding a chemical composition into a compositional descriptor. Using A_2_BCO_5_ as an example, the tensor embeddings of the end members are weighted based on their compositional ratios (AO:BO:CO_2_ = 2:1:1). The mean, standard deviation, and covariance between columns are then calculated and arranged to form the compositional descriptor.

In this study, we developed a CRC prediction model using a systematic approach. The overview of this method is shown in Fig. 1. First, pseudo-binary oxide data were transformed into tensor-type representations of the end members and their composition ratios, and Tucker decomposition was applied to derive tensor embeddings for the end members. Next, chemical compositions were encoded into vector representations (compositional descriptors) using statistical features, such as the mean and standard deviation of the tensor embeddings, as described in the method shown in Fig. 2. A prediction model was then trained exclusively on pseudo-binary oxide compositions. The relationships learned by the model were subsequently evaluated through correlation analysis of the tensor embeddings of the end members. Using this trained model, we predicted the existence probabilities of pseudo-ternary and pseudo-quaternary oxide compositions. Our approach’s success in predicting multi-component compositions from pseudo-binary data indicates its potential for advancing the exploration of other anionic systems, particularly those with fewer known multi-component compounds^25^.

## Results

### Tensor embeddings of end members via tucker decomposition

**Fig. 3:**
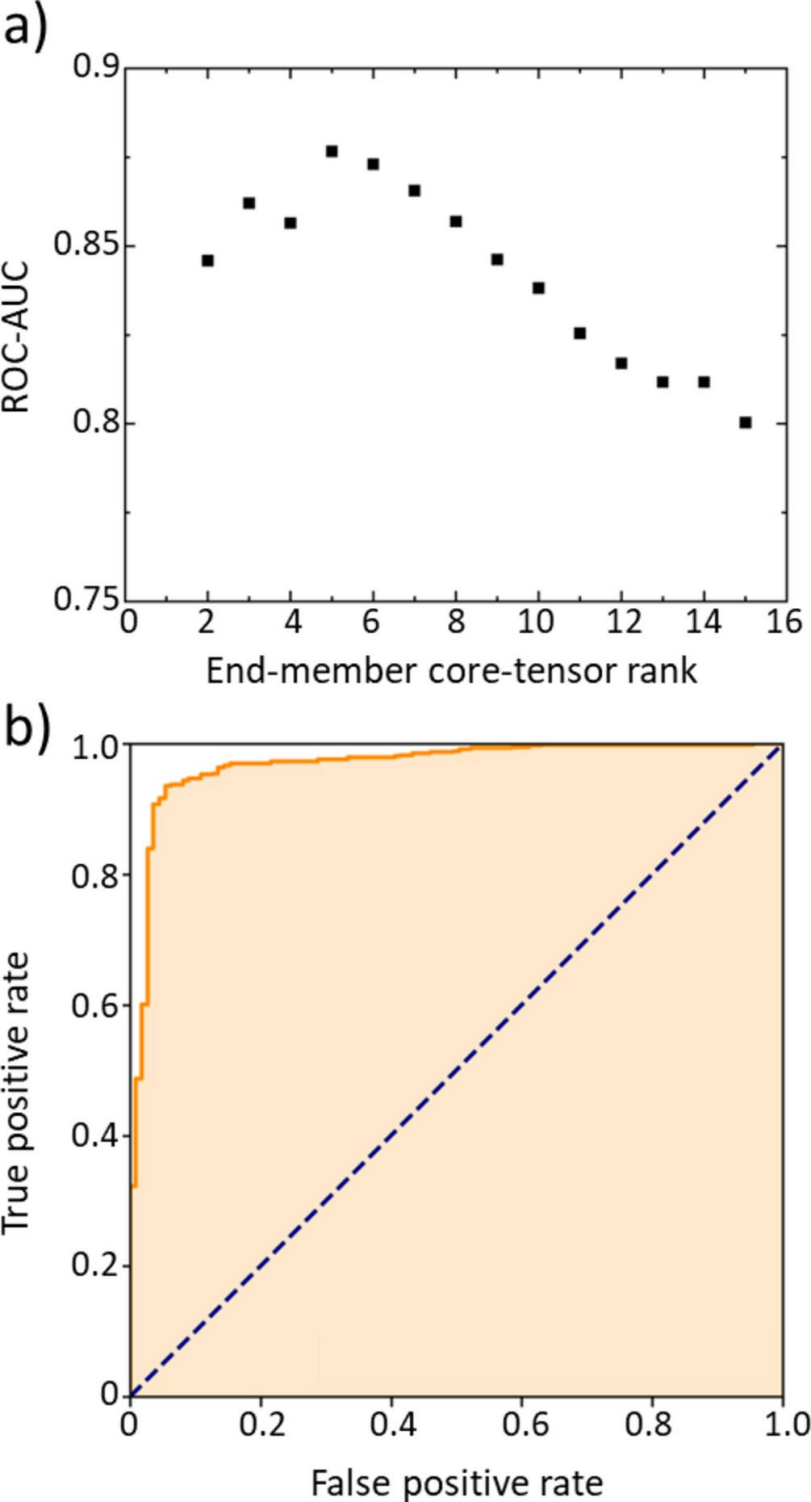
(**a**) ROC-AUC performance as a function of the core tensor rank of the end members in Tucker decomposition cross-validation, with the compositional ratio rank fixed at 7. (**b**) ROC curves for the cases where the core tensor ranks of the end members and compositional ratios are 5 and 7, showing the highest ROC-AUC values.

As shown in Fig. 3a, the Receiver Operating Characteristic-Area Under the Curve (ROC-AUC) in cross-validation varies based on the rank of the core tensor assigned to the end members. The ROC-AUC reached a maximum value of 0.88 when the core tensor rank was set to 5, confirming that the masked data within the tensor could be accurately reproduced. Figure 3b shows the ROC curve at the optimal core tensor rank, indicating that the sharp rise at the high-score side (lower left of the ROC curve) demonstrates superior prediction performance for high scores. By determining the core tensor rank in this manner, the dimensionality of the embedding vectors for the end members was set to 5.

**Fig. 4:**
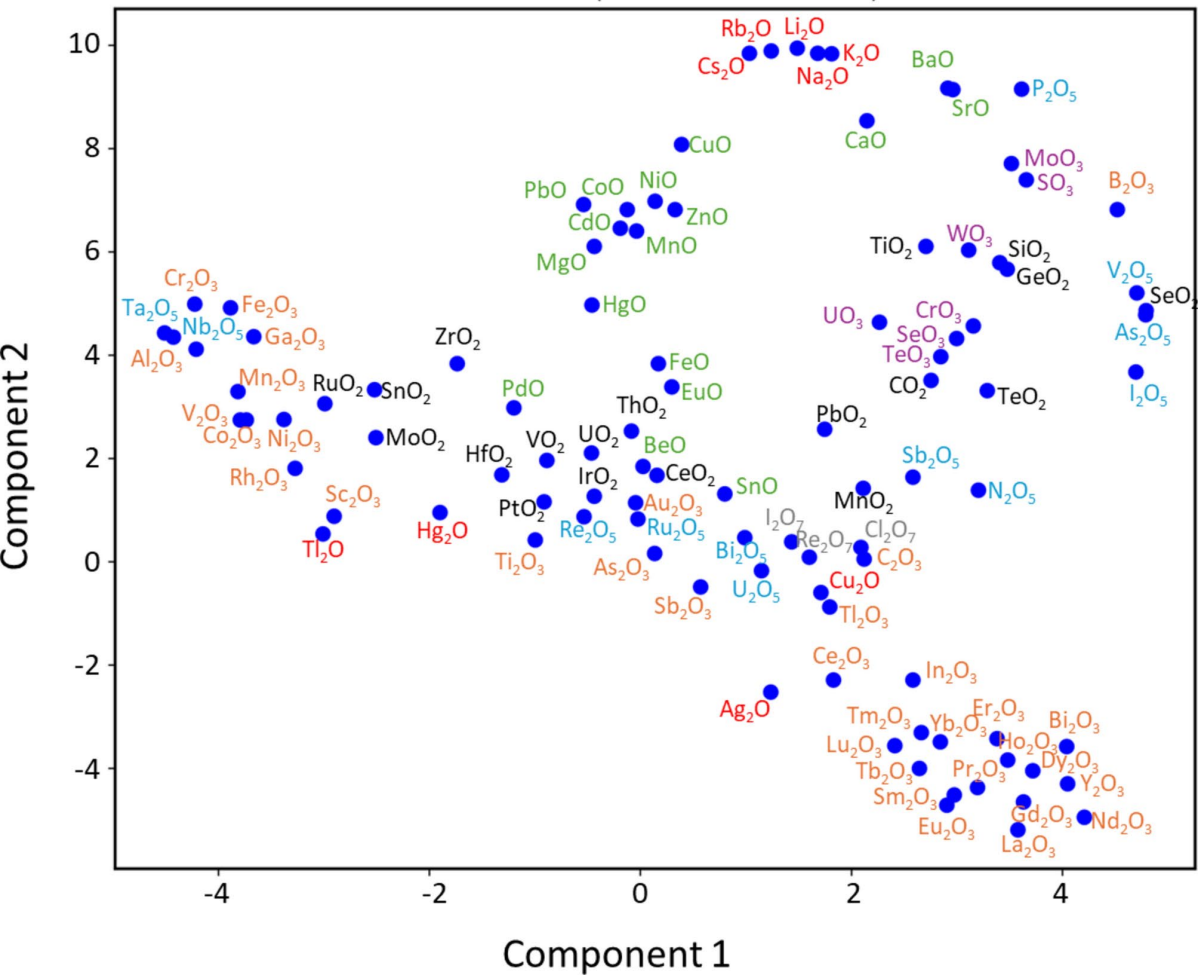
Two-dimensional t-SNE plot of 5-dimensional tensor embeddings for end members, showing clusters based on oxidation states. The end members are color-coded based on their formal oxidation states: red for monovalent (A_2_O), green for divalent (AO), orange for trivalent (A_2_O_3_), black for tetravalent (AO_2_), cyan for pentavalent (A_2_O_5_), purple for hexavalent (AO_3_), and gray for heptavalent (A_2_O_7_).

Figure 4 shows a plot of the end members’ tensor embeddings reduced from 5 to 2 dimensions using t-Distributed Stochastic Neighbor Embedding (t-SNE) based on cosine distance^30^. It was confirmed that the end members were clustered together based on their oxidation states, and within the same oxidation state, alkali metal oxides, Group 11 oxides like Cu_2_O and Ag_2_O, as well as oxides from the sixth period such as Hg_2_O and Tl_2_O, were located close to each other. Additionally, for trivalent end members, 4f. rare earth metal oxides, 3*d* transition metal elements, and Group 13 element oxides were also found to be in proximity, suggesting that chemical features other than oxidation states were also captured.

### Prediction of pseudo-ternary and quaternary oxide compositions using random forest classifier

**Fig. 5:**
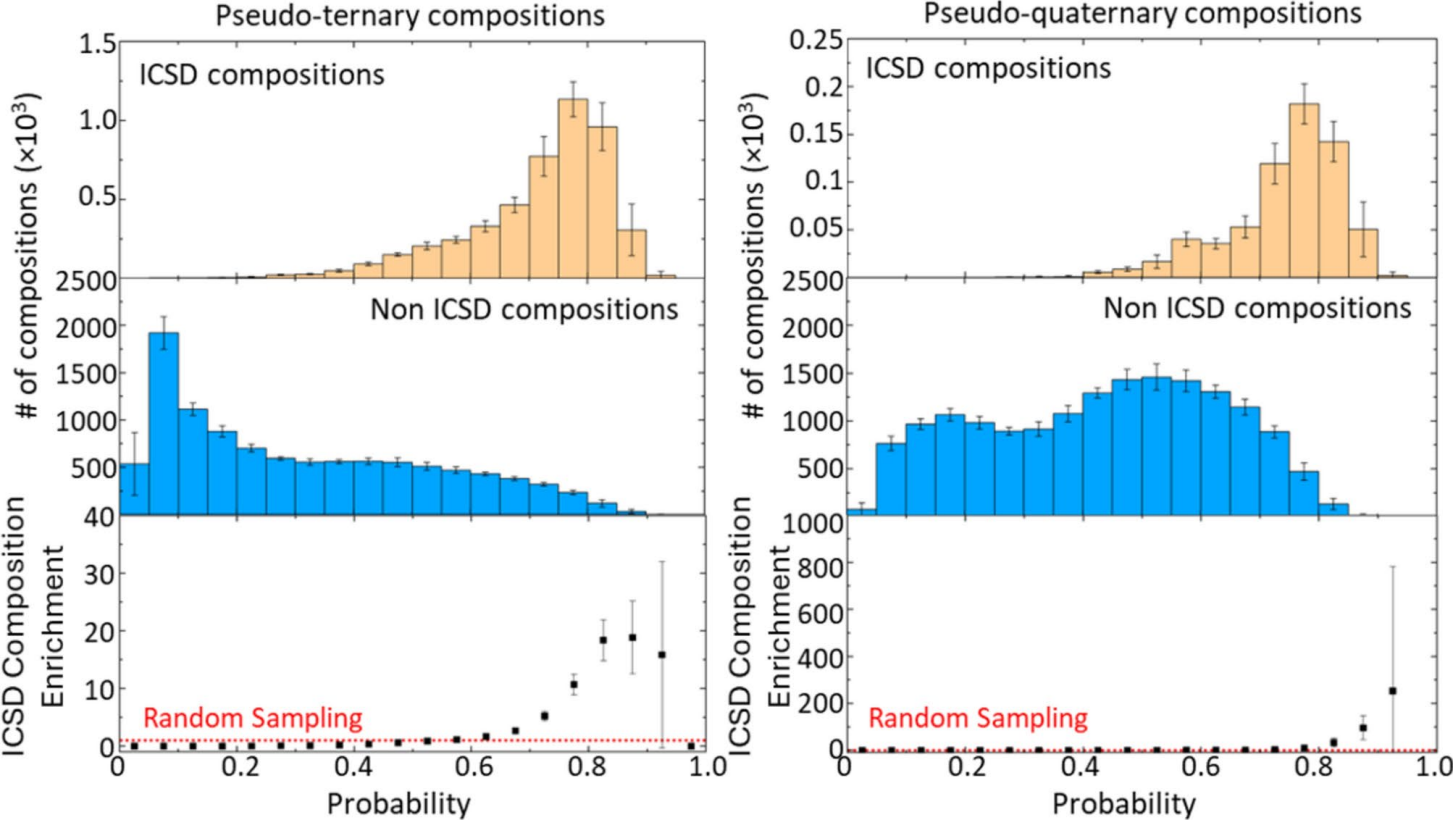
The top row shows histograms of ICSD compositions, and the middle row shows histograms of non-ICSD compositions. The bottom row represents the ratio of ICSD compositions in each bin, normalized by the ratio obtained from random sampling. The left panel presents the results for pseudo-ternary oxide compositions, while the right panel shows the results for pseudo-quaternary oxide compositions. The bin width for the histograms is 0.05.

By applying a Random Forest classifier to the tensor embeddings of the end members, we trained a model on pseudo-binary oxide compositions, and the probabilities of existence of pseudo-ternary and quaternary oxide compositions were predicted. Figure 5 shows the distribution of predicted values for compositions registered in the Inorganic Crystal Structure Database (ICSD). The predicted values for ICSD compositions were concentrated in the high-score region. On the other hand, the peak probability for pseudo-ternary oxide compositions that were not registered in the ICSD was below 0.1, while for pseudo-quaternary compositions, the peak was around 0.55, indicating the presence of many compositions across a wide range of probabilities. This suggests that the dataset used for learning, which was limited to relatively simple pseudo-binary oxide compositions, may be insufficient to represent the complexity of more intricate end member combinations. Furthermore, the probability distributions for pseudo-ternary and quaternary oxide compositions registered in the ICSD were found to be similar, indicating that adding data for pseudo-ternary compositions to the training set could potentially improve the prediction accuracy for pseudo-quaternary compositions. The bottom histogram shows the proportion of ICSD compositions within each bin, normalized against random sampling, allowing for an evaluation of the predictive model’s performance for each probability. For pseudo-ternary oxide compositions, the model outperformed random sampling in regions with probabilities exceeding 0.6, with up to a 19-fold improvement. For pseudo-quaternary oxide compositions, performance exceeded random sampling for probabilities above 0.8, reaching a maximum improvement of 250-fold.

**Fig. 6:**
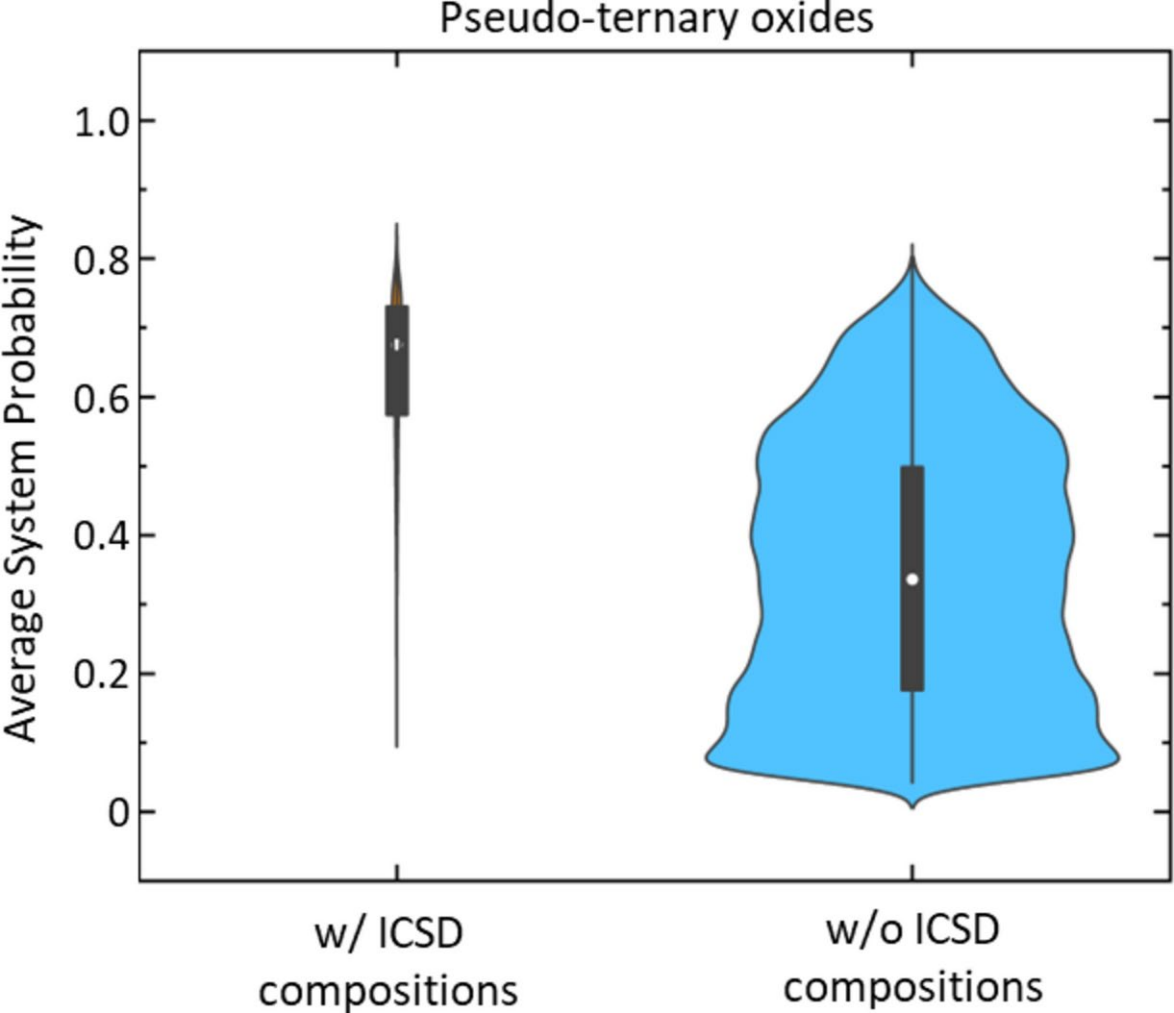
Violin plots of average system probabilities for pseudo-ternary oxide systems, comparing systems with (left) and without (right) ICSD compositions. The black boxes represent the interquartile range, and the white dots indicate the mean values.

For unregistered compositions, the number of compositions exceeding the probability threshold was 1,558,737 for pseudo-ternary systems and 120,863 for pseudo-quaternary systems, both of which far exceed the synthetic capabilities of a traditional laboratory. However, it should be noted that this vast number includes compositions that are only slightly different from those with the highest scores. Moreover, in practical synthesis, there is a possibility of obtaining the desired novel composition even with slight deviations in composition. Therefore, selecting systems with high synthetic feasibility and prioritizing the synthesis of compositions that exhibit maxima within those systems can significantly improve the efficiency of exploration. Figure 6 shows the distribution of the average probability across all compositions within a pseudo-ternary system for 166,650 pseudo-ternary systems (i.e., _101_C_3_), divided based on whether they contain ICSD compositions. The 3656 systems containing ICSD compositions have higher average system probabilities compared to the 162,994 systems without ICSD compositions. This indicates that using the average system probability for each system is effective in selecting systems with a higher likelihood of containing synthesizable compositions. The upper quartile of the average system probability containing ICSD compositions is 0.73, and only 0.5% of the systems without ICSD compositions have an average system probability higher than this value. These systems, in particular, are promising candidates for synthesizing novel materials.

## Discussion

In our approach, the pseudo-binary oxide compositions listed in the ICSD were converted into data capturing the end members and their compositional ratios. Using Tucker decomposition, we then derived tensor embeddings for these end members. The tensor embeddings successfully captured chemical trends, including oxidation states and periodic table positions, demonstrating that Tucker decomposition can automatically extract relevant chemical knowledge without additional input. Furthermore, by using these tensor embeddings, the complex oxide compositions were encoded into vector form, and the model trained only on pseudo-binary oxide compositions was used to evaluate the feasibility of pseudo-ternary and quaternary oxides. As a result, many known materials registered in the ICSD exhibited high probabilities of existence, whereas hypothetical oxide compositions that were not registered tended to have lower probabilities. Additionally, systems containing known compositions had higher average scores compared to those containing only unknown compositions, suggesting that selecting systems with higher average scores could enhance the efficiency of exploration in synthetic experiments. These results demonstrated that even a model trained solely on pseudo-binary oxide compositions could predict the compositions of more complex pseudo-ternary and quaternary oxides. Moreover, the proposed method is expected to facilitate efficient exploration of less-explored multi-component compounds, such as sulfides and nitrides, compared to oxides^25^.

## Methods

### Data preprocessing

We sourced chemical composition data from the Inorganic Crystal Structure Database (ICSD, version 2023)^31^. The target data were selected based on the following criteria: First, the pseudo-*N*-component chemical compositions must contain *N* distinct cations, and the formal charges must be registered as integers. Additionally, the anions were restricted to oxide ions (O^2−^), and the ratios of constituent elements had to be integers. We also required that a prototype structure be registered, and that the composition differed from the prototype structures of the *N*−1 or fewer components to exclude solid solutions. This study exclusively utilizes the compositional information provided by the ICSD, without direct consideration of crystal structures, XRD patterns, or single-phase conditions. Our approach focuses solely on the chemical validity of compositions. The cations targeted are shown in Table 1. These 101 cations appeared at least 15 times in pseudo-binary oxides; those with fewer occurrences were excluded to maintain prediction accuracy and avoid unnecessary expansion of the search space. When converting pseudo-binary oxides into tensor data, the composition ratios were adjusted to simple integer ratios by dividing the mole fractions of the end members into 11 segments and assigning the median of each segment as the representative mole ratio. This approach ensured that the tensor did not become excessively sparse. Consequently, the number of pseudo-binary, pseudo-ternary, and pseudo-quaternary oxide compositions amounted to 3182, 4807, and 660, respectively.

**Table 1:** Metal elements and their formal oxidation states in the target chemical compositions registered in the ICSD database.

Valence	Elements
1	Ag Cs Cu Hg K Li Na Rb Tl
2	Ba Be Ca Cd Co Cu Eu Fe Hg Mg Mn Ni Pb Pd Sn Sr Zn
3	Al As Au B Bi C Ce Co Cr Dy Er Eu Fe Ga Gd Ho In La Lu Mn Nd Ni Pr Rh Sb Sc Sm Tb Ti Tl Tm V Y Yb
4	C Ce Ge Hf Ir Mn Mo Pb Pt Ru Se Si Sn Te Th Ti U V Zr
5	As Bi I N Nb P Re Ru Sb Ta U V
6	Cr Mo S Se Te U W
7	Cl I Re

Among the constituent cations of selected pseudo-binary oxides, those that appear 15 or more times are extracted.

### Creation of tensor embeddings for end members

Pseudo-binary oxide compositions were first enumerated to establish a dataset, capturing the primary compositional characteristics necessary for tensor representation. This enumeration enabled the assignment of consistent tensor embeddings, reflecting underlying chemical trends and periodic relationships within the pseudo-binary oxide systems. For example, MgAl_2_O_4_ was represented as both [MgO, AlO_1.5_, 1:2] and [AlO_1.5_, MgO, 2:1], while SrTiO_3_ was represented as both [SrO, TiO_2_, 1:1] and [TiO_2_, SrO, 1:1]. In these representations, while Al^3+^ would typically correspond to Al_2_O_3_, we adjusted the representation so that the number of cations was always 1. Since the order of the end members does not hold specific significance, both sequences were considered. These end members, along with the composition ratios, were represented as third-order tensor data. For each element of the tensor, if a known pseudo-binary composition existed, we assigned 2 points; if the end members were identical, we assigned 1 point (as no pseudo-binary composition exists), and otherwise, we assigned missing values. Tucker decomposition was applied to this tensor data using the Tensorly module^32^, and the rank of the core tensor was determined through Bayesian optimization with the Optuna module^33^, utilizing tenfold cross-validation (CV) and receiver operating characteristic (ROC) curve along with the area under the curve (ROC-AUC) scores^30^.

### Encoding of feature vectors for pseudo-*N*-component oxide compositions

Using the tensor embeddings for the end members, we weighted them by their composition ratios and calculated statistical features, including the mean, standard deviation, and covariance between columns. For pseudo-ternary and pseudo-quaternary systems, the molar fractions were varied in increments of 0.1 and 0.2, respectively. By aggregating these statistical quantities, we encoded the feature vectors for the chemical compositions. The total number of independent compositions for pseudo-binary, pseudo-ternary, and pseudo-quaternary oxides were 55,550 (computed as _101_C_2_ × 11), 5,999,400 (computed as _101_C_3_ × 36), and 16,331,700 (computed as _101_C_4_ × 4), respectively.

### Construction of a prediction model using a random forest classifier

To construct the prediction model, we employed only pseudo-binary oxide compositions for which tensor embeddings were generated, using these as training data and assigning 2 points for positive examples. For negative examples, in addition to combinations of two identical end members as used in Tucker decomposition, we randomly selected 10% of the 52,368 (= 55,550 − 3182) unregistered combinations in ICSD. The Random Forest Classification was implemented using the Scikit-learn module^30^. This process was repeated 10 times with different selections, and the average value was utilized as the prediction score for multi-component compositions. The model’s hyperparameters (i.e., the number of decision trees and the maximum depth) were tuned using Bayesian optimization, employing tenfold CV and ROC-AUC scores. Utilizing the optimized parameters, we assessed the distribution of predicted scores for both known and hypothetical pseudo-ternary and pseudo-quaternary oxide compositions.

## Data Availability

The data supporting the findings of this study are available from the corresponding author upon reasonable request. The code used in this study will be made publicly available in a GitHub repository (https://github.com/hirhay/TensorEmbeddings4CRC/tree/main) upon acceptance of the manuscript. The repository includes all necessary code, documentation, and instructions to ensure reproducibility.
